# Mechanical Strain Regulates Osteoblast Proliferation through Integrin-Mediated ERK Activation

**DOI:** 10.1371/journal.pone.0035709

**Published:** 2012-04-23

**Authors:** Yu-xian Yan, Yuan-wei Gong, Yong Guo, Qi Lv, Chun Guo, Yan Zhuang, Yuan Zhang, Ruixin Li, Xi-zheng Zhang

**Affiliations:** 1 Institute of Medical Equipment, Academy of Military Medical Science, Tianjin, China; 2 Experiment Management Center of Medical College of People's Armed Police Forces, TianJin, China; University of Sassari, Italy

## Abstract

Mechanical strain plays a critical role in the proliferation, differentiation and maturation of bone cells. As mechanical receptor cells, osteoblasts perceive and respond to stress force, such as those associated with compression, strain and shear stress. However, the underlying molecular mechanisms of this process remain unclear. Using a four-point bending device, mouse MC3T3-E1 cells was exposed to mechanical tensile strain. Cell proliferation was determined to be most efficient when stimulated once a day by mechanical strain at a frequency of 0.5 Hz and intensities of 2500 µε with once a day, and a periodicity of 1 h/day for 3 days. The applied mechanical strain resulted in the altered expression of 1992 genes, 41 of which are involved in the mitogen-activated protein kinase (MAPK) signaling pathway. Activation of ERK by mechanical strain promoted cell proliferation and inactivation of ERK by PD98059 suppressed proliferation, confirming that ERK plays an important role in the response to mechanical strain. Furthermore, the membrane-associated receptors integrin β1 and integrin β5 were determined to regulate ERK activity and the proliferation of mechanical strain-treated MC3T3-E1 cells in opposite ways. The knockdown of integrin β1 led to the inhibition of ERK activity and cell proliferation, whereas the knockdown of integrin β5 led to the enhancement of both processes. This study proposes a novel mechanism by which mechanical strain regulates bone growth and remodeling.

## Introduction

Wolff's law describes the relationship between bone morphology and mechanical load. The regular load is important for maintaining the integrity of bone [1]. The mechanical load ensures that the bone is constantly updated itself and that any internal structural defects are repaired. Disuse or a lack of load caused by events, such as prolonged bed rest, spinal cord injury or space flight, results in the rapid loss of bone mass and even osteoporosis in some cases [2]. Conversely, overloaded strain leads to pathological bone modeling, remodeling, or microdamage that may result in fracture when accumulated [3]–[6].

Mechanical loads include mechanical strain and compressive and shear stresses. The mechanical microenvironment within a tissue can influence the fate of a cell. Such local mechanical stimuli result in mechanotransduction, which is the conversion of a physical signal into intracellular biochemical cascade signals [7]–[11]. Previous studies have shown how mechanics can be transformed into chemical signals due to changes in protein conformation and the presentation of previously cryptic binding sites [12]. Other studies have confirmed that these events could potentially alter gene expression, protein activity and ultimately cell function [9], [11]. Mechanical strain has been reported to induce bone remodeling activity resulting in structural changes. This type of stimulation can promote the proliferation and anabolism of osteoblasts in order to facilitate bone tissue reconstruction, contributing to the homeostasis of bone tissue [13]–[19]. In bone, mechanical stimuli are transmitted through the extracellular matrix (ECM) to resident osteoblasts, osteocytes, periosteal cells and osteoclasts [20]. Osteoblasts are important mechanical receptors that can transform mechanical stimuli into biochemical signals and secrete bone matrix to promote bone matrix mineralization [21]. However, how cells convert the mechanical signal into a biological signal and relaying the signaling pathway to regulate cell proliferation remain to be unfully elucidated.

Previous studies have demonstrated that integrins function as mechanotransducers. Matziolis and colleagues reported that the expression of integrin β1 increased 2.2- fold following mechanical stimulation [22]. Kasten and colleagues applied drag forces to integrin β1 on the apical surface of adherent human MSC and confirmed that the expression of vascular endothelial growth factor (VEGF) and collagen I were induced by integrin β1-mediated mechanical forces, which are involved in osteogenesis [23]. Additionally, studies have demonstrated that the expression of integrin α5β1 was reduced after skeletal unloading caused by hind limb elevation [24], [25]. These studies showed that integrins, which are receptors for mechanical loading in bone, form an important link between the extracellular matrix and the cytoskeleton, transducing mechanical signals imposed on bone into responses from bone cells.

Biomechanical signals are essential for bone homeostasis, growth, adaptation, healing and remodeling [14], [26]–[29]. Mechanical forces have been shown to activate many types of signal transduction cascades, including the MAPK signal pathway [30]. The role of MAPK signaling components, such as extracellular signal-regulated kinase (ERK), c-Jun N-terminal kinase (JNK), and p38 MAPK (p38), have been shown to favor osteoblastic cell proliferation and differentiation [31]–[33]. In particular, ERK1/2 is involved in cell transformation, proliferation, and the survival of several cell types, including osteoblasts [34]. However, the mechanism by which cells convert a mechanical signal into a biological signal has not been fully elucidated.

In this study, we investigated the effects of mechanical strain on mouse MC3T3-E1 cell proliferation. A microarray analysis was used to investigate gene expression profiles in cells under mechanical strain. Based on those results, we focused on exploring the involvement of MAPK signaling pathways. Our results demonstrate that integrins β1-and β5-mediated ERK signaling affected cell proliferation in response to mechanical strain. This study revealed the importance of mechanical stimulation in bone growth and remodeling as well as its underlying molecular mechanism.

## Results

### Mechanical strain promotes proliferative activity in MC3T3-E1 cells

**Figure 1:**
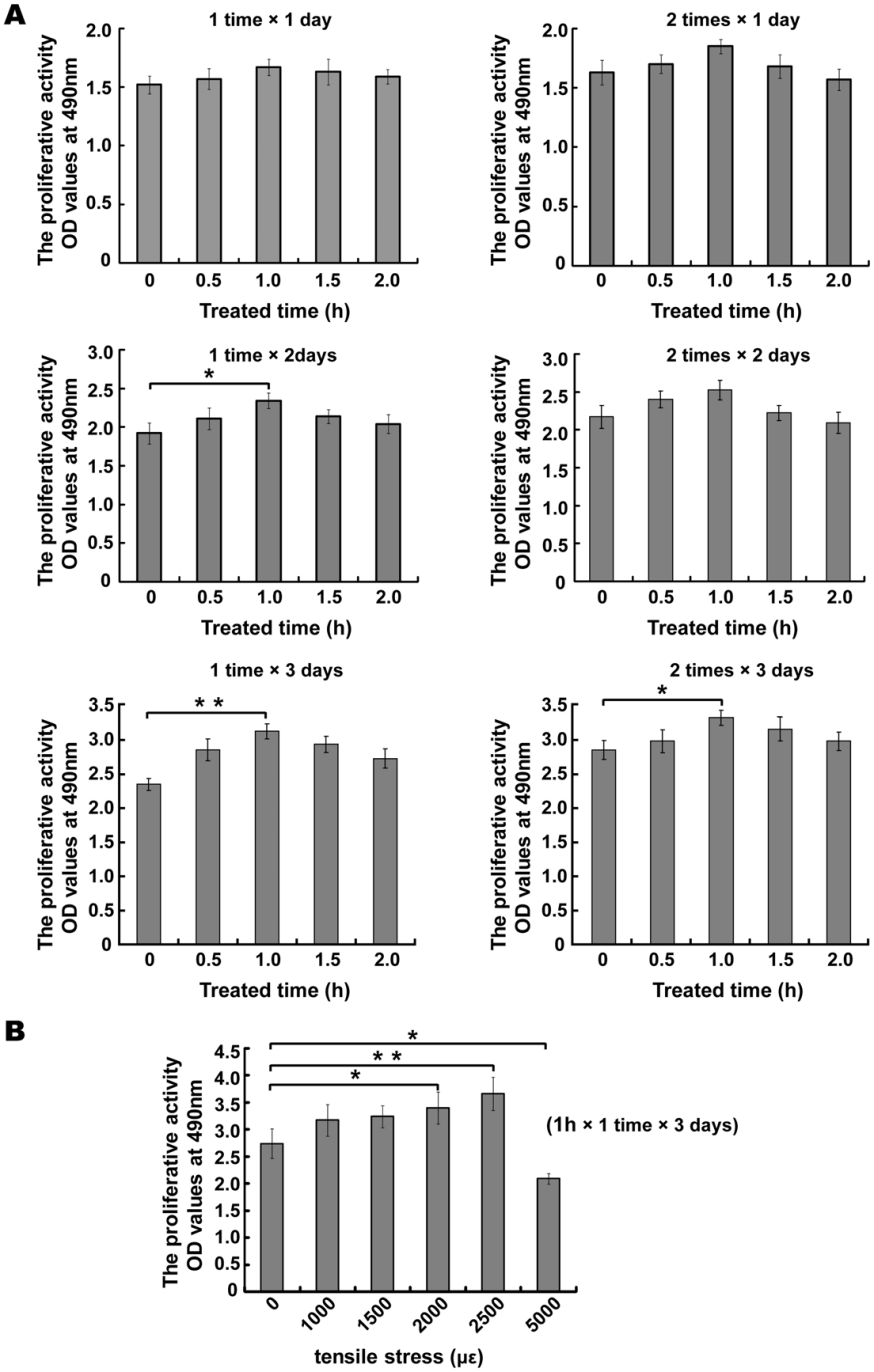
The best efficiently stimulation of mechanical strain was determined by MTT in MC3T3-E1 cells. (A) The proliferation was evaluated by MTT assay under mechanical strains of 2500 µε at 0.5 Hz with different strain time (0.5 h–2.5 h) and periodicity (once or twice a day) and strain cycle (1 day–3 day). Data are represented as the mean ± SD of at least three biological replicates, *P<0.05, **P<0.01 compared with treat time (0 h) group. (B) The proliferation was evaluated by MTT assay in different intensities (1000–5000 µε) at 0.5 Hz, once a day for 1 hour over 3 consecutive days. Data are represented as the mean ± SD of at least three biological replicates, * *P*<0.05, ** *P*<0.01 compared with treat time (0 h) group.

Mechanical strain is important for cell proliferative activity. The influence of strain on the proliferative activity of MC3T3-E1 cells was tested using different strain times and frequencies. The following initial test conditions were used: a working tensile strain of 2500 µε, which is within the physiological range, applied once or twice a day; a strain cycle of 1 day and strain times of 0.5, 1, 1.5, 2 and 2.5 h. There was no significant difference observed with respect to the proliferation rates among the different groups. When the strain cycle was increased from 1 to 2 days, a 1 h strain time resulted in increased proliferative activity. However, there was no obvious difference when the number of strains per day increased from 1 to 2. On the basis of these results, the following conditions resulted in the highest cell proliferation rate: a 2500 µε strain applied once a day for 1 hour over 3 consecutive days (Fig. 1A). Cell proliferative activity under the following different mechanical strains was examined: 1000, 1500, 2000 and 2500 µε, which are all within the physiological range; and 5000 µε, which is above physiological range. Strains of 2000 and 2500 µε markedly promoted cell proliferation, whereas a strain of 5000 µε inhibited cell proliferation (Fig. 1B). The mechanical strain of 5000 µε enhanced PI positive stained percent and lactate dehydrogenase (LDH) activity in the culture medium of the cells, indicating that the strain of 5000 µε resulted in cell necrosis and overloading, which is unsuitable for cell growth (Figure S1, and S2).

### Microarray analysis of gene expression profile in MC3T3-E1 cells under mechanical strain

**Figure 2:**
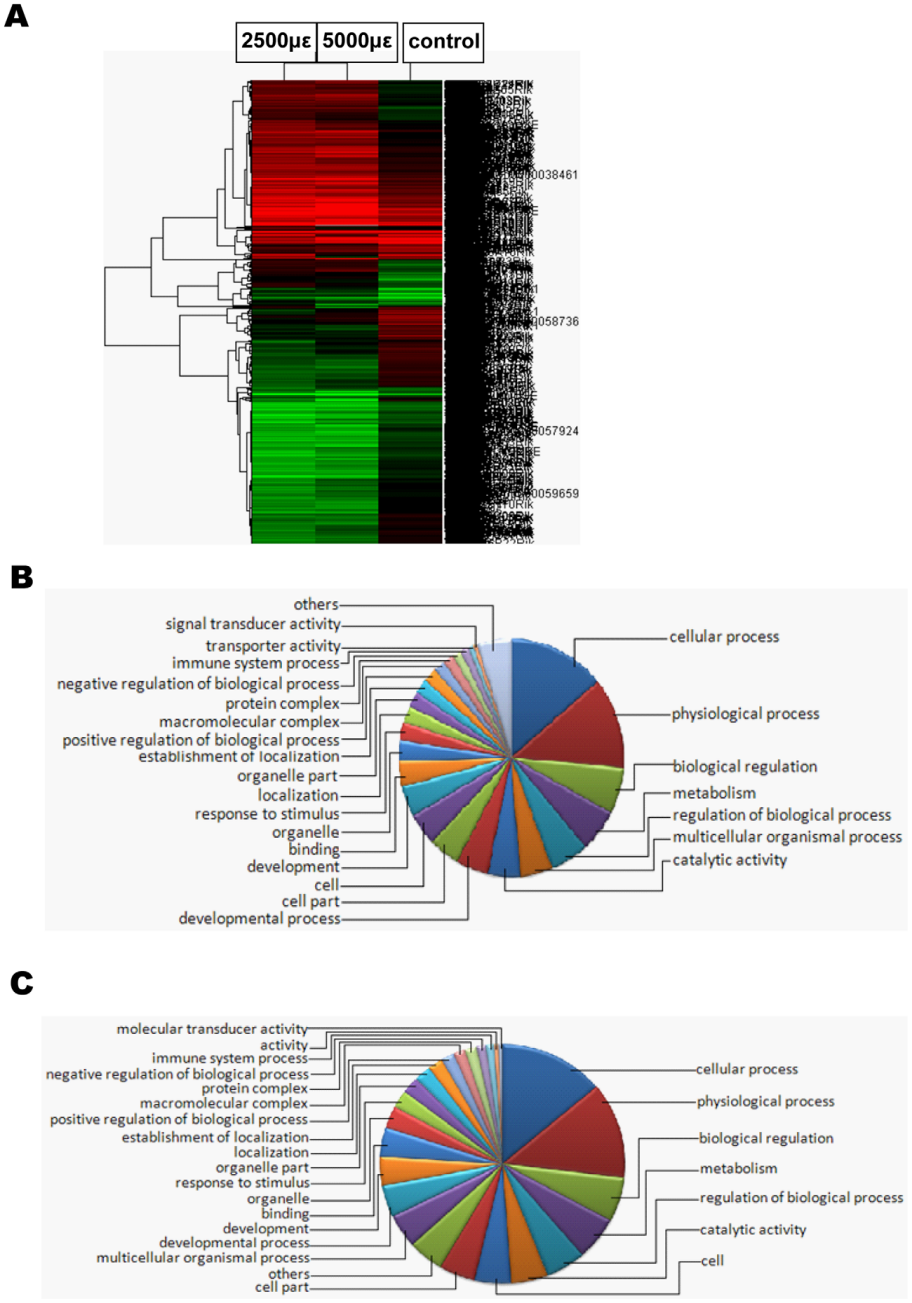
Microarray analysis of gene expression profile in MC3T3-E1 cells under mechanical strain. (A) Hierarchical clustering analysis with the heat map under 2500 and 5000 µε at 0.5 Hz applied once a day for 1 h over 3 consecutive days in MC3T3-E1 cells. Each row represents a gene; red: up-regulated genes, Green: down-regulated genes. (B) Clustering map in gene function of MC3T3-E1 cells when stimulated by mechanical strain of 2500 µε at 0.5 Hz applied once a day for 1 h over 3 consecutive days. (C) Clustering map in gene function of MC3T3-E1 cells when stimulated by mechanical strain of 5000 µε at 0.5 Hz applied once a day for 1 h over 3 consecutive days.

The CapitalBio microarray platform, which contains 32,000 mouse genes, was validated by the MicroArray Quality Control project initiated by the US Food and Drug Administration. To determine the effects of mechanical strain on gene expression, MC3T3-E1 cells were subjected to the following mechanical strain conditions: 2500 and 5000 µε at 0.5 Hz applied once a day for 1 h over 3 consecutive days. Compared with the control group, MC3T3-E1 cells under a strain of 2500 µε exhibited significantly different expression levels of 1992 genes, with 776 genes being expressed at levels more than 2-fold higher and 1216 genes expressed at 0.5-fold lower levels (Fig. 2A). The majority of these genes were assigned to 24 functional groups (Fig. 2B). MC3T3-E1 cells treated with a strain of 5000 µε exhibited significantly different expression levels for 1435 genes, with 737 genes having higher expression levels and 698 genes having lower expression levels compared those of the control group (Fig. 2A). The majority of these genes were assigned to 24 functional groups (Fig. 2C). Further statistical analyses revealed that the 2500 µε load affected 45 signaling pathways, including the MAPK signaling pathway as well as ECM-receptor interactions, axon guidance and antigen processing and presentation. Information of microarray analyses the main signaling pathways in MC3T3-E1 cells with mechanical strain is stated in Table 1.

**Table 1:** Microarry analyses the main signaling pathways in MC3T3-E1 cells with mechanical strain.

KEGG Pathway Name	Total	P-value	Q-value
MAPK signaling pathway	40	0.0	0.0
Regulation of actin cytoskeleton	40	0.0	0.0
Focal adhesion	38	0.0	0.0
Ribosome	36	0.0	0.0
Tight junction	33	0.0	0.0
Cell cycle	32	0.0	0.0
Axon guidance	31	0.0	0.0
Insulin signaling pathway	30	0.0	0.0
Calcium signaling pathway	27	0.0	0.0
Wnt signaling pathway	22	4.0E-6	9.0E-6
Purine metabolism	22	4.0E-6	9.0E-6
Cytokine-cytokine receptor interaction	22	0.001297	0.002261
Pyrimidine metabolism	21	0.0	0.0
Natural killer cell mediated cytotoxicity	21	5.2E-5	1.12E-4
T cell receptor signaling pathway	21	0.0	0.0
Gap junction	19	0.0	0.0
Glutathione metabolism	19	0.0	0.0
Leukocyte transendothelial migration	19	4.2E-5	9.2E-5
Cell adhesion molecules (CAMs)	19	0.0	0.0
Oxidative phosphorylation	18	3.88E-4	7.55E-4
VEGF signaling pathway	18	2.0E-6	5.0E-6
Olfactory transduction	17	0.0	0.0
Neuroactive ligand-receptor interaction	17	0.300574	0.319936
B cell receptor signaling pathway	16	3.0E-6	7.0E-6
mTOR signaling pathway	16	0.0	1.0E-6
Apoptosis	16	1.8E-5	4.1E-5
TGF-beta signaling pathway	15	1.38E-4	2.87E-4
Antigen processing and presentation	15	0.0	0.0
DNA polymerase	15	0.0	0.0
Jak-STAT signaling pathway	14	0.00545	0.00866
Long-term depression	13	6.99E-4	0.001287
Metabolism of xenobiotics by cytochrome P450	13	0.0	0.0
Cell Communication	13	0.066975	0.092911
Toll-like receptor signaling pathway	13	3.53E-4	6.98E-4
ECM-receptor interaction	12	0.00222	0.003727
Adherens junction	12	6.99E-4	0.001287
Phosphatidylinositol signaling system	12	3.5E-5	7.9E-5
Type I diabetes mellitus	12	0.0	0.0
Tyrosine metabolism	11	0.003309	0.005458
Alanine and aspartate metabolism	11	0.0	0.0
Glycolysis/Gluconeogenesis	10	0.001357	0.002317
Urea cycle and metabolism of amino groups	9	9.0E-6	2.2E-5
Protein export	6	0.0	0.0
Retinol metabolism	5	0.0	0.0
Pentose and glucuronate interconversions	3	0.0	0.0

### The effects of mechanical strain on the MAPK signaling pathway and associated gene expression

**Figure 3:**
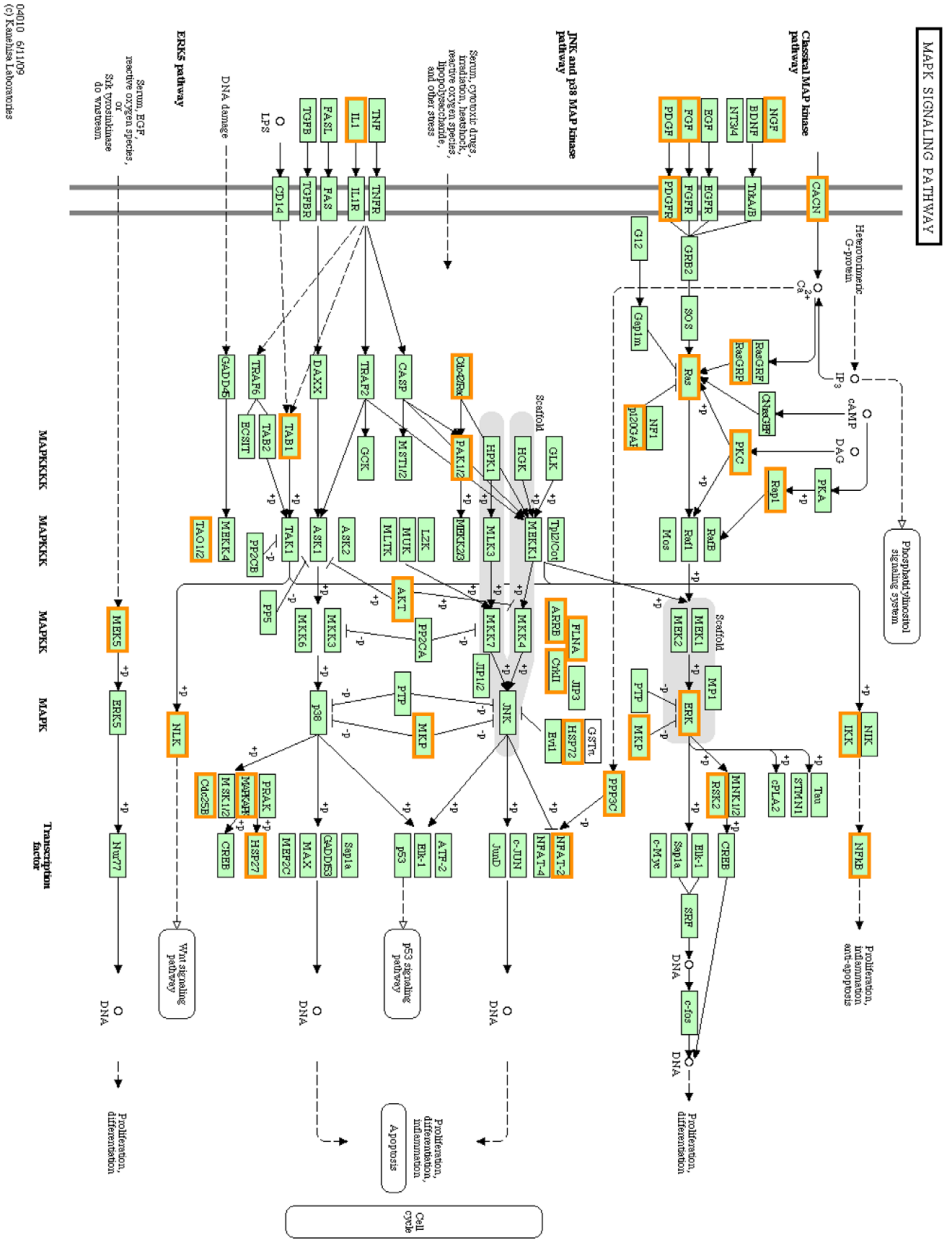
The KEGG MAPK signaling pathway showing genes differentially expressed in MC3T3-E1 cells exposed to mechanical strain. Mechanical strains of 2500 µε, once a day at 0.5 Hz, and a periodicity of 1 h/day for 3 days. Orange frame indicates changed genes. KEGG, Kyoto Encyclopedia of Genes and Genomes.

**Figure 4:**
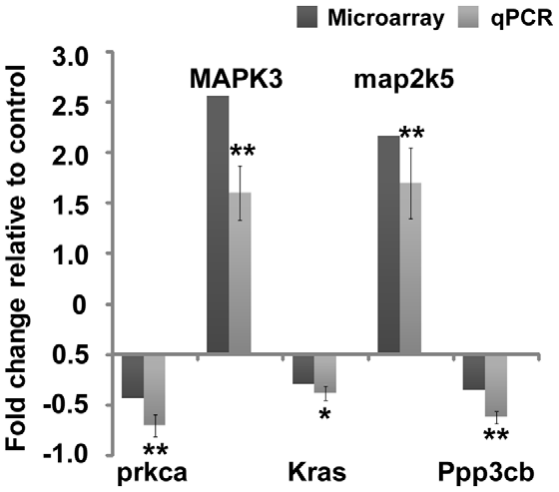
Effect of mechanical strain on the MAPK signaling pathway. The expression values of the selected 5 genes from the MAPK signaling pathway was measured by cDNA microarray and quantitative real-time RT-PCR (qRT-PCR). MC3T3-E1 cells were stimulated under mechanical strains of 2500 µε, once a day at 0.5 Hz, and a periodicity of 1 h/day for 3 days. Data are represented as the mean ± SD of at least three biological replicates, * *P*<0.05, ** *P*<0.01.

The expression of 41 genes involved in MAPK pathway was altered in response to mechanical strain, with 16 genes higher and 25 genes lower expression levels (Fig. 3, Table 2). Bioinformatic analysis revealed that the MAPK signaling pathway was significantly involved in the response to the 2500 µε mechanical strain (Fig. 3). Furthermore, the changes observed in the expression levels of 5 genes involved in the MAPK signaling pathway were confirmed using real-time PCR (Fig. 4), supporting the observation that 2500 µε of mechanical strain affected the MAPK signaling pathway.

**Table 2:** The changed genes of the MAPK signaling pathway under mechanical strain of 2500 µε, at 0.5 Hz, once a day for 1 h over 3 consecutive days in MC3T3-E1 cells.

Gene	Ratio	Ref Seq	Gene description
Hspb1	5.6516	-	heat shock protein 1
Rras	3.0868	NM_009101	“Harvey rat sarcoma oncogene, subgroup R
Cacnb3	3.0815	NM_007581	calcium channel, voltage-dependent, beta 3 subuni
Map3k7ip1	3.0340	NM_025609	mitogen-activated protein kinase kinase kinase 7 interacting protein 1
Rac3	2.9686	NM_133223	RAS-related C3 botulinum substrate 3
Nfkb2	2.8445	NM_019408	nuclear factor of kappa light polypeptide gene enhancer in B-cells 2, p49/p100
Mapk3	2.563	NM_0119521346859	mitogen activated protein kinase 3
Pdgfa	2.4456		platelet derived growth factor, alpha
Akt2	2.3886	NM_007434	thymoma viral proto-oncogene 2
Map2k5	2.3626	NM_011840	mitogen activated protein kinase kinase 5
Flnc	2.3287	XM_284175	filamin C, gamma (actin binding protein 280)
Mras	2.3192	NM_008624	muscle and microspikes RAS
Hspa2	2.3179	NM_008301	heat shock protein 2
Dusp1	2.0974	NM_013642	dual specificity phosphatase 1
Akt1	2.0353	NM_009652	thymoma viral proto-oncogene 1
Nlk	0.4764	NM_008702	nemo like kinase
Cdc25b	0.4696	NM_023117	cell division cycle 25 homolog B (S. cerevisiae)
Mapkapk3	0.4678	-	mitogen-activated protein kinase-activated protein kinase 3
Kras	0.4665	NM_021284	v-Ki-ras2 Kirsten rat sarcoma viral oncogene homolog
Crk	0.4654	NM_133656	v-crk sarcoma virus CT10 oncogene homolog (avian)
Rasa1	0.4495	NM_145452	RAS p21 protein activator 1
Rap1b	0.4458	NM_024457	RAS related protein 1b
Rras2	0.4392	NM_025846	related RAS viral (r-ras) oncogene homolog 2
Prkca	0.4350	NM_011101	“protein kinase C, alpha
Fgf7	0.4309	NM_008008	fibroblast growth factor 7
Cacna1b	0.4196	XM_486780	
Il1a	0.4167	NM_015747	solute carrier family 20, member 1
Dusp3	0.4080	-	peptidyl prolyl isomerase H
Ppp3cb	0.3522	NM_008914	protein phosphatase 3, catalytic subunit, beta isoform
Rasgrp1	0.3346		
Nfatc2	0.3323	NM_011960	poly (ADP-ribose) glycohydrolase
Pak2	0.3316		p21 (CDKN1A)-activated kinase 2
Arrb2	0.3233	NM_026218	
Cacnb2	0.3124	0.0	
Chuk	0.3036	NM_007700	conserved helix-loop-helix ubiquitous kinase
Nras	0.2953	AC163297	neuroblastoma ras oncogene
Bdnf	0.2927	-	similar to high mobility group protein (LOC382421), misc RNA
Pdgfrb	0.2353	NM_008809	“platelet derived growth factor receptor, beta polypeptide
Rps6ka6	0.2291	XM_488517	
Taok1	0.1552		

### Mechanical strain promotes MC3T3-E1 cell proliferation through the ERK signaling pathway

**Figure 5:**
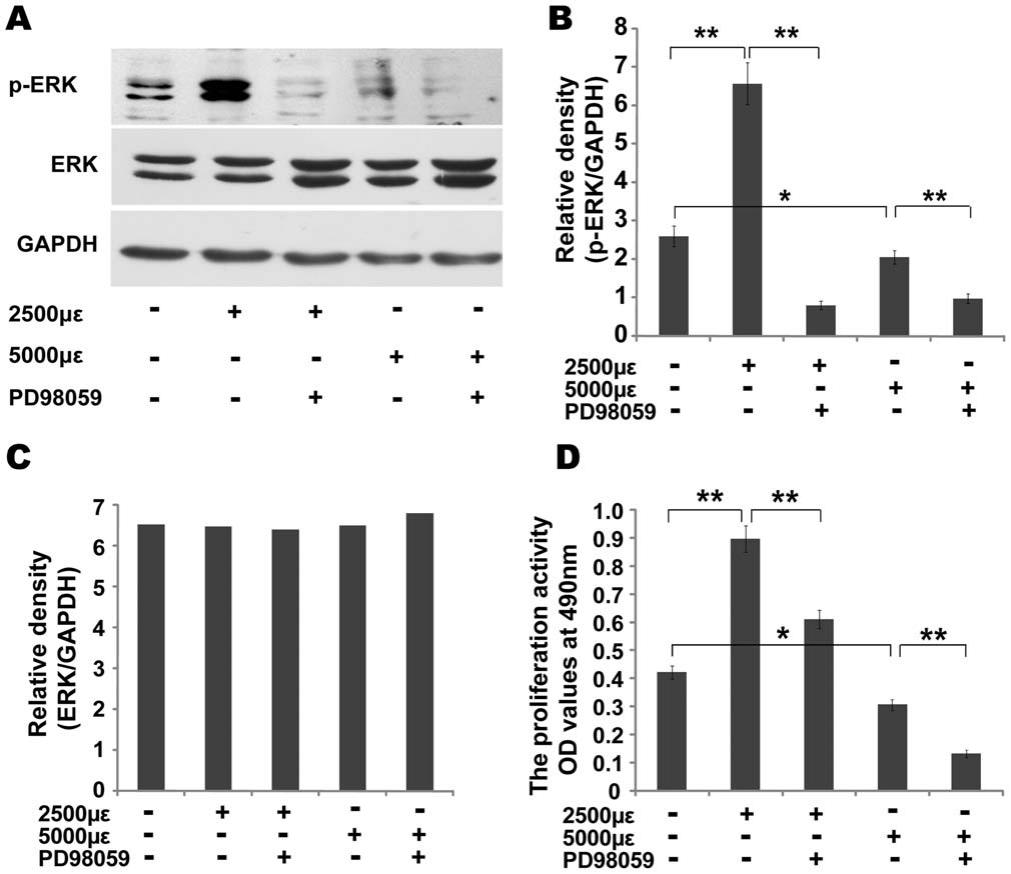
Mechanical strain promotes MC3T3-E1 cells proliferation through the ERK signaling pathway. (A–C) The protein expression of ERK and ERK-phosphorylation in different treat groups (with or without 20 µM MEK1/2 inhibitors PD98059) was detected by Western blotting with anti-ERK1/2 and anti-p-ERK1/2. GAPDH was used as an internal control. Data are represented as mean ± SD of at least three biological replicates, * *P*<0.05, ** *P*<0.01. (D) The proliferation of cells treated with or without PD98059 under mechanical strain was evaluated by MTT assay. Data are represented as the mean ± SD, of at least three biological replicates, * *P*<0.05, ** *P*<0.01.

Given that ERK is an important type of MAPK, we examined its role in MC3T3-E1 cell proliferation while under mechanical strain. MC3T3-E1 cells were exposed to periodic mechanical strains of 2500 and 5000 µε, respectively, once a day at 0.5 Hz, and a periodicity of 1 h/day for 3 days. After the exposure of cells to 2500 µε of mechanical strain, ERK was activated, as its degree of phosphorylation sharply increased and the cell proliferation rate was significantly increased (Fig. 5). In contrast, 5000 µε of strain inactivated ERK, as the degree of ERK phosphorylation sharply decreased and the cell proliferation rate was significantly reduced (Fig. 5). In addition, specific chemical inhibitors of ERK1/2 were used to determine whether the mechanical strain affected MC3T3-E1 cell proliferation through the ERK signaling pathway. The MEK1/2 inhibitor PD98059 selectively prevents the activation of MEK1/2 by Raf and subsequently blocks the ERK1/2 cascade. We pretreated MC3T3-E1 cells with PD98059 (20 µM) or DMSO (0.1%, solvent control) for 30 min before applying mechanical strain. The cells were then subjected to mechanical strains of 2500 and 5000 µε once a day at 0.5 Hz and a periodicity of 1 h/day for 3 days. Compared to unstrained (control) cells or cells that were under only mechanical strain, phosphor-ERK was significantly decreased in pretreated cells and significantly inhibited 2500 µε-induced effects on cell proliferation. These results indicate that physiological mechanical strain promotes the activation of MC3T3-E1 cell proliferation and that mechanical strains above physiological strain decreased MC3T3-E1 cell proliferation, with both occurring through the ERK signaling pathway (Fig. 5).

### Mechanical strain induces the expression of integrins β1 and β5

**Figure 6:**
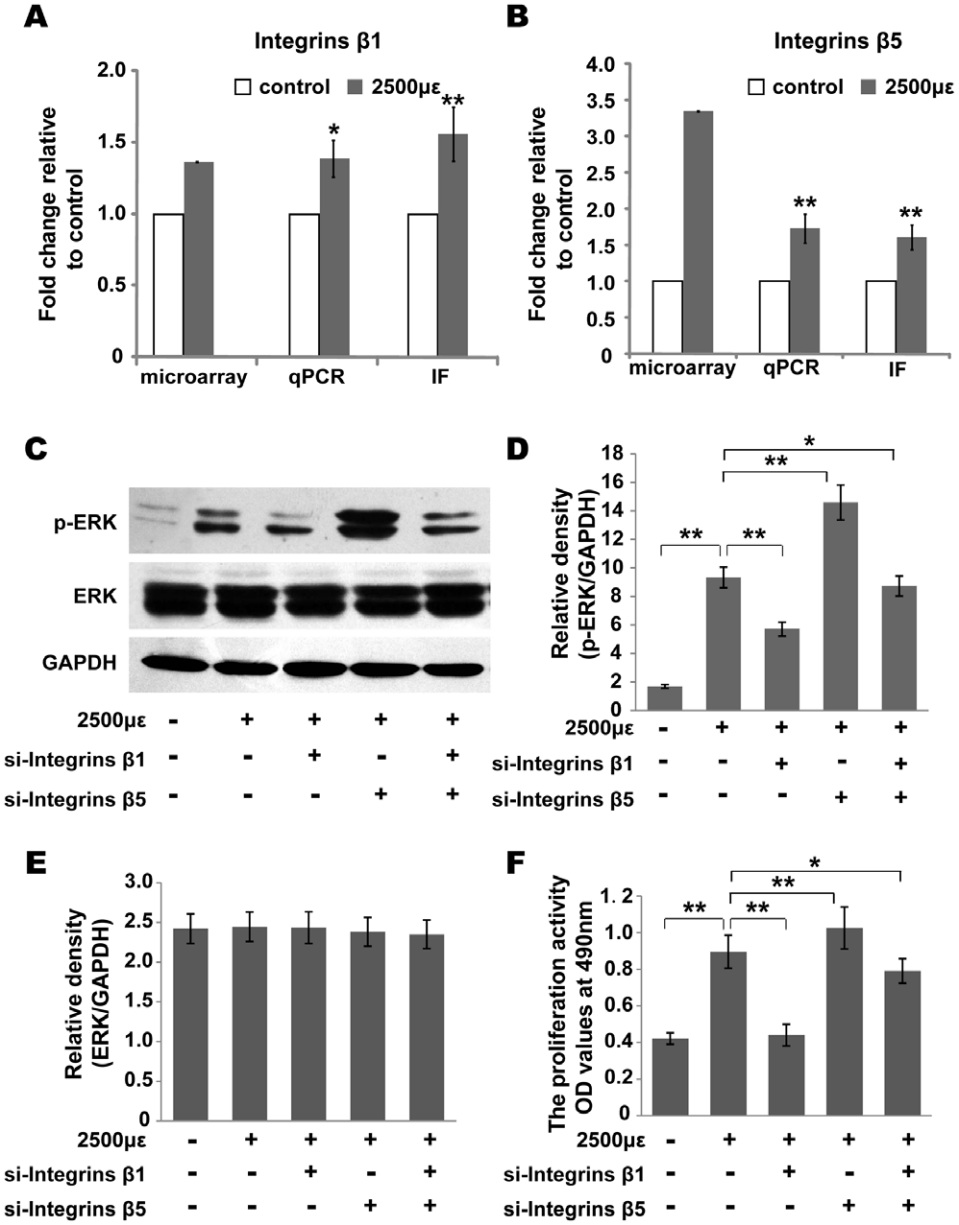
Integrin β1 and Integrin β5 have opposite effects on the phosphorylation of ERK and the proliferation in MC3T3-E1 cells. (A) and (B) Mechanical strain induces Integrin β1 and Integrin β5 expression. The mRNA and protein expressions of Integrin β1 (A) and Integrin β5 (B) were analysised by cDNA microarray, qPCR and immunofluorescence (IF) with anti-Itgb1 and anti-Itgb5. All data are represented as mean ± SD of at least three biological replicates. * *P*<0.05, ** *P*<0.01 versus Control. (C–E) The protein expressions of ERK and ERK-phosphorylations in different treated groups (knockdown of Integrinβ1, Integrinβ5 or both simultaneously with siRNA transfection) under mechanical strain were detected by Western blotting with anti-ERK1/2 and anti-p-ERK1/2. GAPDH was used as an internal control. All data represent the mean ± SD of at least three biological replicates, * *P*<0.05, ** *P*<0.01 between the indicated groups. (F) The proliferation of cells treated with Integrin β1-siRNA, Integrin β5-siRNA or both under mechanical strain was evaluated by MTT assay. All data represent the mean ± SD of at least three biological replicates, * *P*<0.05, ** *P*<0.01 between the indicated groups.

Previous studies have confirmed that integrins are mechanical stimulus-specific receptors and different integrin subtypes have different effects. After MC3T3-E1 cells were exposed to periodic mechanical strain, microarray analysis revealed that the mRNA expression levels of integrins β1 and β5 increased by 1.36- and 3.35- fold, respectively. After the exposure of cells to 2500 µε of strain, real-time PCR results showed that the expression levels of integrins β1 and β5 increased 1.39 and 1.73 -fold, respectively, compared with those of the control group. Furthermore, immunofluorescence analysis confirmed that the expression of both integrins β1 and β5 exhibited the same changes. Altogether, these results demonstrate that exposure to the 2500 µε of mechanical strain causes the gene and protein expression of integrins β1 and β5 to increase compared with that of the control group (Fig. 6 A–B).

### Integrins β1 and β5 are involved in ERK activation and cell proliferation

To study the role of integrins in ERK activation and cell proliferation, RNA interference was used to knockdown the expression of Integrins β1 and β5. After siRNA knockdown of Integrins β1 and β5, the mRNA and protein expression levels of Integrins β1 and β5 significantly decreased compared with those in cells under only 2500 µε of mechanical strain (Figure S3). When cells were exposed to 2500 µε of mechanical strain, the effects of the knockdown of integrins β1, β5 or both simultaneously on cell proliferation and ERK activity were examined. The knockdown of integrin β1 lowered the phosphorylation level of ERK and cell proliferation rate. In contrast, the knockdown of integrin β5 significantly increased ERK phosphorylation and the cell proliferation rate. In the cells in which both integrins β1 and β5 were knocked down, pERK levels and the cell proliferation rates were significantly higher than those in cells with only integrin β1 knocked down and lower than those in cells with only integrin β5 knocked down (Figure 6 C, D, E, F). In unstrained cells, the effects of knocking down integrins β1, β5 or both of them simultaneously on ERK activation and cell proliferation were similar to those of mechanically strained cells (Figure S4 and S5). However, the phosphorylation levels of ERK were much lower than those of strained cells. These results indicate that integrin β1 Integrin β5 are involved in MC3T3-E1 cell proliferation activity in response to mechanical strain in a manner that is mediated by the ERK signaling pathway, with the two integrins exhibiting opposite effects (Fig. C, D, E, F).

## Discussion

The purpose of this study was to investigate the mechanism of mechanical strain regulating the proliferation of MC3T3-E1 cells. Our results showed that 2500 µε of mechanical strain applied once a day at 0.5 Hz and a periodicity of 1 h/day for 3 days significantly promotes the proliferation of MC3T3-E1 cells. This mechanical strain induces changes in multiple intracellular signaling pathways. The MAPK pathway is an important mechanical signal transduction pathway in which ERK plays a major role. The ERK pathway mediates the mechanical strain-induced proliferation of MC3T3-E1 cells. More importantly, the mechanical receptors integrins β1 and β5 exhibit opposite effects in the regulation of ERK activation and cell proliferation. These results indicate that mechanical strain regulates osteoblast proliferation via integrin β1/β5-mediated ERK activation.

The MAPK signaling pathway, which is one of the mechanical signaling pathways identified by our microarray analysis, is a signal transduction pathway closely related to mechanics and plays an important role in regulating cell proliferation [35]. A mechanical stimulus was first reported to activate the MAPK signaling pathways in skeletal muscle [35]. Kusumi and colleagues [36] applied 7%, 0.25 Hz cyclic tensile strain (CTS) applied to osteoblasts for 4 h a day over 3 consecutive days and demonstrated that p38 MAPK and ERK1/2 were competitively activated, suggesting that the p38 MAPK pathway can regulate the ERK1/2 pathway in osteoblasts under CTS. In addition, Hatton JP and colleagues found that short periods of mechanical stress induced early gene expression and growth in MC3T3-E1 osteoblasts, primarily through an ERK1/2-mediated pathway [37].

The ERK pathway plays a key role in osteoblast proliferation but has little effect on osteoblast differentiation [34], [38]–[40]. ERK is mainly activated through Ras-Raf-MEK1/2-ERK via phosphorylation. Because only phosphorylated ERK has catalytic activity, this study investigated the changes in ERK1/2 and its associated activity in response to different mechanical strains by determining the p-ERK/ERK ratio using western blot analysis.

This study shows that a 2500 µε of mechanical strain applied once a day at 0.5 Hz and a periodicity of 1 h/day for 3 days can increase ERK phosphorylation and cell proliferation in MC3T3-E1 cells. PD98059, an antagonist of MEK1/2, which blocks ERK activation during the application of 2500 µε of mechanical strain, leads to a significant decrease in cell proliferation. This observation indicates that mechanical strain can transform extracellular mechanical signals into biological signals through the ERK signaling pathway, affecting cell proliferation.

Integrins represent a major family of cell-surface receptors that are transmembrane heterodimers comprised of noncovalently bound α- and β-subunits [41]. Each subunit contains a large extracellular domain, a transmembrane region and a small intracellular region. The extracellular domain directly interacts with ECM proteins, including fibronectin, laminin, collagen and vitronectin. The intracytoplasmic area interacts with intracellular signal transmission molecules and cytoskeletal proteins to regulate cell functions, such as signal transduction, cytoskeletal remodeling, cell motility, migration, apoptosis, cell proliferation and cell adhesion [42], [43]. As the main receptors that connect the cytoskeleton to the extracellular matrix (ECM), integrins have an intimate relationship with mechanical strain. Integrins provide a preferred site for mechanical signal transfer across the cell surface and transmit the signal across the plasma membrane and to the cytoskeleton over a specific molecular pathway [44]. Integrins act as mechanical transduction receptors and the stimulation of these receptors has been shown to modulate cellular growth and gene expression [45]. In addition, integrins act as mechanical strain receptors in bone and transduce mechanical signals imposed on the bone into responses from bone cells [46], [47]. However, but the precise molecular basis for this regulation remains unclear.

Integrin β1 has been reported to be expressed on the surface of osteoblasts [48] and mechanical stimulation can cause redistribution [49] of integrin β1 on the cell surface. Integrin β1 antibodies inhibit the activity of mechanosensitive ion channels, demonstrating that β1 integrin can mediate the impact of mechanical strain on bone. Hu and colleagues [50] directly imposed an external force on integrins with a three-dimensional magnetic distortion device, providing a simple and effective method to study the transmembrane transfer of external force. In their experiments, physical distortion forces acting directly on β1 integrin in endothelial cells resulted in effective transmembrane force transmission, demonstrating that integrin β1 can transmit external forces to the cytoskeleton. Using molecular dynamics simulations of the FNIII_10_-αvβ3 integrin complex, Vogel revealed that simulated mechanical force or “manual” opening of the hinge accelerated formation of the T-junction induced by ligand binding and hinge opening. These results provided a common structural model for the dynamic process by which integrins become activated [51].

Carvalho and colleagues [49] confirmed that mechanical stress upregulated the expression of β1 integrin in osteosarcoma cells. Another investigation confirmed [52] that a 30 min steady fluid shear stress of 20 dynes/cm^2^ increased the expression of the following early mechanoresponsive genes: integrin β1 in C57BL/6J mouse osteoblasts, Wnt, estrogen receptor, insulin-like growth factor-I, and bone morphogenetic protein. In addition, integrin β1 was required for the focal adhesion kinase (FAK) independent activation of MAP kinases (ERK1/2, p38 and JNK) induced by mechanical stress [53]. α_v_β_3_ and β_1_ integrins mediated the proliferation of human osteoblast-like MG63 cells induced by oscillatory shear stress [54]. However, the effect of cyclic tensile strain on MC3T3-E1 cell proliferation activity through the integrin β1-mediated ERK signaling pathway has rarely been reported. Moreover, there are only a few studies on the effects of integrin β5 on bone cells. Xianghong Luan and colleagues [55] indicated that the posteruptive axial movement of teeth involves the significant formation of a new apical root cementum and alveolar bone in tandem with the upregulation of collagen I, integrin β5, and SPARC (osteonectin) gene expression. However, the effects of integrin β5 on osteoblast cells under the mechanical strain have not been reported.

In this study, the expression at the mRNA and protein levels of integrins β1 and β5 were observed to increase under mechanical strain. After knockdown of integrin β1, ERK phosphorylation and cell proliferation significantly decreased. ERK phosphorylation and cell proliferation under a mechanical strain of 2500 µε were lower in the absence of integrin β1 than in the presence of integrin β1. In contrast, knockdown of integrin β5 resulted in a significant increase in the levels of ERK phosphorylation and cell proliferation. ERK phosphorylation and cell proliferation under a mechanical strain of 2500 µε were higher in the absence of integrin β5 than in the presence of integrin β5. These data demonstrated that mechanical strain could affect the ERK activity-mediated proliferation of MC3T3-E1 cells via integrins β1 and β5, with these two integrins having opposite effects. In addition, in the absence of both integrins, ERK phosphorylation and cell proliferation were significantly higher than in the absence of only integrin β1 and lower in the absence of only integrin β5. These observations indicate that the absence of both integrins has a superimposed effect, further illustrating the opposing roles that integrins β1 and β5 play in the regulation of MC3T3-E1 cell proliferation via the ERK signaling pathway.

In summary, our study demonstrates that mechanical strain can regulate osteoblast proliferation through the integrin β1/β5-mediated ERK signaling pathway. In the signaling pathway of mechanotransduction, the two integrins have opposite effects; integrin β1 facilitate mechanotransduction and integrin β5 obstruct it. This study is the first showing how mechanical strain promotes the proliferation of osteoblast cells via ERK mediation through integrins β1 and β5.

## Materials and Methods

### Materials

TRIzol was purchased from Invitrogen (Invitrogen, USA). The DNA microarray used in this study was produced by CapitalBio Corporation (Beijing, China). MTT was purchased from Solarbio. SYBR Green Master Mix kits were purchased from Applied Biosystems (Applied Biosystems, USA). Rabbit monoclonal antibodies (mAb) against ERK1/2 (#4695) and phospho-ERK1/2 (#4376) were purchased from Cell Signaling Technology (CST), and mAb against integrin β1 (#1798-1) was obtained from Epitomics. Integrin β5 antibody (AB1926) was purchased from Millipore. Goat anti-rabbit FITC-conjugated IgG (H+L) (sc-bsF-0295G) was purchased from Bioss (Biosynthesis Biotechnology, BEIJING). Control small interfering RNA (siRNA), specific siRNAs for integrins β1 and β5, Lipofectamine 2000 and fluorescent oligomers were purchased from Invitrogen (Carlsbad, CA, USA). PD98095 (sc-10006726) was purchased from Cayman (Cayman Chemical, USA). All other chemicals of reagent grade were obtained from Sigma unless otherwise noted.

### Cell culture

The mouse osteoblastic cell line MC3T3-E1 was purchased from Peking Union Medical College (China, Beijing). Cells were cultivated in α-MEM containing 10% fetal calf serum (Gibco, USA), 100 IU/mL penicillin and 100 µg/mL streptomycin at 37°C in an atmosphere with 5% CO_2_ and 95% humidity, and the medium was exchange every three days. At confluence, cells were digested with 0.25% trypsin and seeded to mechanical load dishes of a four-point bending device for experiments. The ERK1/2 inhibitor PD98059 (20 µM) and DMSO (0.1%) solvent control were added to cell culture 2 hours prior to the application of mechanical strain and remained in the culture media throughout the experiment.

### Mechanical tensile strain

To exert mechanical strain on cells inoculated in mechanical load dishes, we used a previously described four-point bending device [56]–[59] that was independently developed by the Chinese Military Academy of Medical Sciences.

### Cell proliferation

After trypsinized and seeded on mechanical load dishes at a density of 2×10^4^ cells/cm^2^, MC3T3-E1 cells were divided into groups that were then subjected to different mechanical strains. MTT (3-(4, 5-dimethylthiazol-2-yl)-2, 5- diphenyltetrazolium bromide) solution (Promega) was used to assay for living cells after strain was applied to MC3T3-E1 cells. In this assay, MTT is reduced to formazan by intracellular NAD (P) H-oxidoreductase. The formazan product can be detected using an enzyme-linked immunosorbent assay reader at 490 nm. Thus, the absorbance (OD value) at 490 nm was regarded as the relative number of living cells and relative activity of cell proliferation [60].

### Microarray data analysis

Total RNA isolation and assessment: Total cellular RNA was extracted with the Trizol reagent according to the manufacturer's instructions. The RNA concentration and purity of the obtained RNA were determined by OD260/280 nm absorption ratio. The extent of degradation was evaluated by analying the 18S and 28S ribosomal RNAs in formaldehyde cross-linked agarose gels. Only non-degraded RNA samples were used in this study.

cRNA labeling and cDNA synthesis: DNase-treated total RNA (5 µg) was converted to fluorescent dye (Cy5- and Cy3-dCTP)-labeled cDNA using the CapitalBio cRNA Amplification and Labeling Kit (CapitalBio), which employs a modified version of Eberwine's linear RNA amplification method and a subsequent enzymatic reaction [61] Double-stranded cDNA (dsDNA) was then synthesized using DNA polymerase and RNase H. The dsDNA products were purified using the PCR NucleoSpin Extract II Kit (MN) and eluted with 30 µL of elution buffer. The eluted double-stranded cDNA products were vacuum evaporated to a volume of 16 µL, which was used in 40 µL in vitro transcription reactions at 37°C for 4–14 hr using a T7-Oligo (dT) Promoter Primer in the first-strand cDNA synthesis reaction. Amplified cRNA was purified using the RNA Clean-up Kit (MN). The Klenow enzyme labeling strategy was adopted after reverse transcription of the cRNA using CbcScript II reverse transcriptase. The labeled cDNA was purified with a PCR NucleoSpin Extract II Kit (MN) and resuspended in elution buffer. Labeled controls and test samples were quantitatively adjusted on the basis of the efficiency of Cy5- or Cy3-dCTP incorporation and then dissolved in 80 µL of hybridization solution containing 3×SSC, 0.2% SDS, 5×Denhardt's solution and 25% formamide. The DNA in the hybridization solution was denatured at 95°C for 3 min prior to loading onto the microarray. Microarray expression profiling using a 32 k Mouse Genome Array: The arrays were hybridized in a CapitalBio BioMixer™ II Hybridization Station overnight at a rotation speed of 8 rpm and a temperature of 42°C. After washed with two solutions (0.2% SDS, 2×SSC at 42°C for 5 min and 0.2×SSC for 5 min), the arrays were scanned with a confocal LuxScanTM scanner and the resulting images were analyzed using LuxScan™ 3.0 software (both from CapitalBio). For individual channel data extraction, spots with intensities below 400 units after background subtraction in both channels (Cy3 and Cy5) were removed. Space- and intensity-dependent normalization based on the LOWESS program was then employed. To identify genes with significantly different expression levels, Significance Analysis of Microarrays (SAM, version 3.02) was employed. The results were analyzed using multiple bioinformatic methods, including cluster analysis, pathway analysis and GO classification. To confirm the obtained results, 5 differentially expressed genes were selected from the MAPK signaling pathway for Quantitative real-time PCR analysis.

### Quantitative real-time PCR

RNA extraction and quality assessment were performed as described above. RNA samples were subjected to cDNA synthesis, and gene expression analysis was completed using real-time PCR. The total RNA was reverse transcribed in a 20 µL reaction mixture containing 50 ng of oligo-dT primer mix and 2 units of Superscript III reverse transcriptase according to the manufacturer's instructions (Invitrogen). Real-time PCR analysis was performed in an ABI Step-one RealTime PCR machine in a 48-well format (Applied Biosystems, Foster City, CA, USA) using the Fast SYBR-green Master Mix kit (Applied Biosystems, Foster City, CA, USA). The cycling profile was 95°C for 20 s, 95°C for 3 s and 60°C for 30 s for a total of 40 cycles. The reactions were normalized on the basis of the amplification of the selected gene with glyceraldehyde 3-phosphate dehydrogenase (GAPDH) as a control reference. Using the relative quantitative method (2^−ΔΔCT^), the expression levels of the PCR products of interest relative to those in the control group were calculated. The details of the primers are listd in Table S1 of the supporting information.

### Western Immunoblot Assays

Total protein was extracted from the cells and then quantified using the BCA method. For each group, a total of 40 µg of protein was transferred to a NC membrane after electrophoretic separation. The membrane was pre-hybridized in 5 g/L skim milk for 1 h. The appropriate rabbit anti-mouse monoclonal antibody (1∶1000 dilution) was then added, and the membrane was incubated overnight at 4°C. After washing in TBS, the HRP-conjugated goat anti-IgG secondary antibody (1∶1000 dilution) was added, and the membrane was incubated at 37°C for 1 h. After washing in TBS again, the proteins were visualized using an ECL detection kit. GAPDH was used as an internal reference control. Scion Image software was used to perform semi-quantitative analysis.

### Immunofluorescence analysis

After the mechanical stimulation, MC3T3-E1 cells were fixed in 4% paraformaldehyde for 30 min followed by washing in 0.01 M PBS (pH 7.2) for 5 min. After incubation in 0.03% Triton for 20 min and washing 3 times with PBS, the cells were blocked in PBST supplemented with 2% BSA. Next, the rabbit anti-mouse integrins β1 and β5 monoclonal antibodies (1∶200) were incubated with the cells for 72 h at 4°C. The cells were washed 3 times with PBS, and a FITC-conjugated goat anti-rabbit secondary antibody (1∶200) was incubated with the cells for 4 h at room temperature in a dark room. The cells were washed an additional 3 times with PBS. Expression of integrin β1 or β5 was determined using an Infinite 200 (TECAN, Switzerland) fluorescence microplate reader. Integrin β1 or β5 fluorescence intensity was measured according to the excitation/emission at 485/520 nm and given as the mean of eight replicates. The fluorescence background was determined using cells treated with FITC-labeled second antibody.

### siRNA Transfection

After digesting MC3T3-E1 cells in the logarithmic phase as described above, the cells were inoculated in 24-well cell culture plates containing serum- and antibiotic-free α-MEM at a density of 2×10^5^ cells per well. The cells were cultured until they reached 50% confluence and then transfected with a fluorescent oligomer, Stealth siRNA and the Stealth siRNA negative control (Invitrogen) using Lipofectamine 2000 according to the manufacturer's protocol. To determine the best transfection time, the efficiency of the transfection was observed by confocal microscopy at 24, 48, 72 and 96 hours post-transfection. The specific siRNAs for integrins β1 andβ5 (Invitrogen) were transfected using the same method, with siRNA-N again serving as a negative control. Table 3 lists the small RNA sequences. Mechanical strain or pharmacological treatment was applied 48 hours after transfection.

**Table 3:** Small RNA sequences.

Description	Cat No/lot	Type	Sequence	Quantity
Itgb1-MSS205553	10620319/148893 D07	RNA	UAGAAAUGUUGGAACACUUUCGUCC	Stealth
	10620319/148893 D08	RNA	GGACGAAAGUGUUCCAACAUUUCUA	Stealth
Itgb5-MSS205566	10620319/148893 E03	RNA	UACAGCCGCAUGUGCAAUUGUAGGC	Stealth
	10620319/148893 E04	RNA	GCCUACAAUUGCACAUGCGGCUGUA	Stealth

### Statistical analysis

The microarray data were analyzed using LuxScanTM 3.0 software and standardized per spot and per chip by both background subtraction based on negative controls and intensity-dependent normalization (non-linear or LOWESS normalization). The ratio of Cy3 to Cy5 fluorescence was calculated to determine gene expression differences. Cluster analysis was performed using Genespring and Cluster3.0 analysis software. Information on differentially expressed genes was obtained via the United States National Medical Center for Biotechnology Information database (http://www.ncbi.nlm.nih.gov). Signal transduction information analysis was performed using Pathway Studio software. The measurement data are indicated as the mean ± SD. Using the SPSS12.0 statistics package, the means in each group were compared using ANOVA and SNK pairwise comparisons. A *p* value of less than 0.05 was considered to be significant.

## Supporting Information

Figure S1 **Apoptosis percent and PI positive stained percent of MC3T3-E1 cells.** With Apoptosis Assay Kit containing Annexin V labeled with fluorescein isothiocyanate (FITC) and propidium iodide (PI), using fluorescence microscope and flow cytometry, apoptosis percent and PI positive stained percent of MC3T3-E1 cells subjected to different mechanical strain for 3 days were assayed. The apoptosis percent and PI positive stained percent of the cells exposed to 5000 µε were both higher than other groups (0 µε and 2500 µε). The elevation of apoptosis percent was not evident. All data represent the mean ± SD of at least three biological replicates; * *P*<0.05, ** *P*<0.01, between the indicated groups.
(TIF)

Figure S2 **Lactate dehydrogenase (LDH) activity in MC3T3-E1 cells, culture media.** With LDH activity assay Kit, using spectrophotometer, the LDH activity in the cells, culture media was assayed. when MC3T3-E1 cells subjected to different mechanical strain for 3 days, the relative LDH activity in the culture media of the cells exposed to 5000 µε were both higher than other groups (0 µε and 2500 µε). All data represent the mean ± SD of at least three biological replicates, * *P*<0.05, ** *P*<0.01, between the indicated groups.
(TIF)

Figure S3 **Silencing of Integrin β1 or Integrin β5 by specific siRNA respectively.** (**A and B**) The knockdown efficiency of Integrin β1 (A) or Integrin β5 (B) mRNA was verified by qPCR. (**C and D**) The knockdown efficency of Integrin β1 (C) or Integrin β5 (D) protein was verified by immunofluorescence. All data represent the mean ± SD of at least three biological replicates; * *P*<0.05, ** *P*<0.01, between the indicated groups.
(TIF)

Figure S4 **The protein expression of ERK and ERK-phosphorylations in unstrained cells.** The MC3T3-E1 cells, protein levels of ERK and ERK-phosphorylations in different groups {Knockdown of Integrinβ1 (Itgb1), Integrinβ5 (Itgb5) or both simultaneously with siRNA transfection} were detected by Western blotting with anti-ERK1/2 and anti-p-ERK1/2. GAPDH was used as an internal control. All data represent the mean ± SD of at least three biological replicates; * *P*<0.05, ** *P*<0.01, between the indicated groups.
(TIF)

Figure S5 **The proliferative activity of unstrained MC3T3-E1 cells.** The proliferative activity of the cells in different groups {Knockdown of Integrinβ1 (Itgb1), Integrinβ5 (Itgb5) or both simultaneously with siRNA transfection} were detected by MTT assay. All data represent the mean ± SD of at least three biological replicates; * *P*<0.05, ** *P*<0.01, between the indicated groups.
(TIF)

Table S1 **Sequences of primers used for qRT–PCR.**
(DOC)
