# Supplementary figures and images for: Mechanical Strain Regulates Osteoblast Proliferation through Integrin-Mediated ERK Activation
Source: PLoS One. 2012 Apr 23;7(4):e35709. doi: 10.1371/journal.pone.0035709 (PMC3335094; doi:10.1371/journal.pone.0035709)

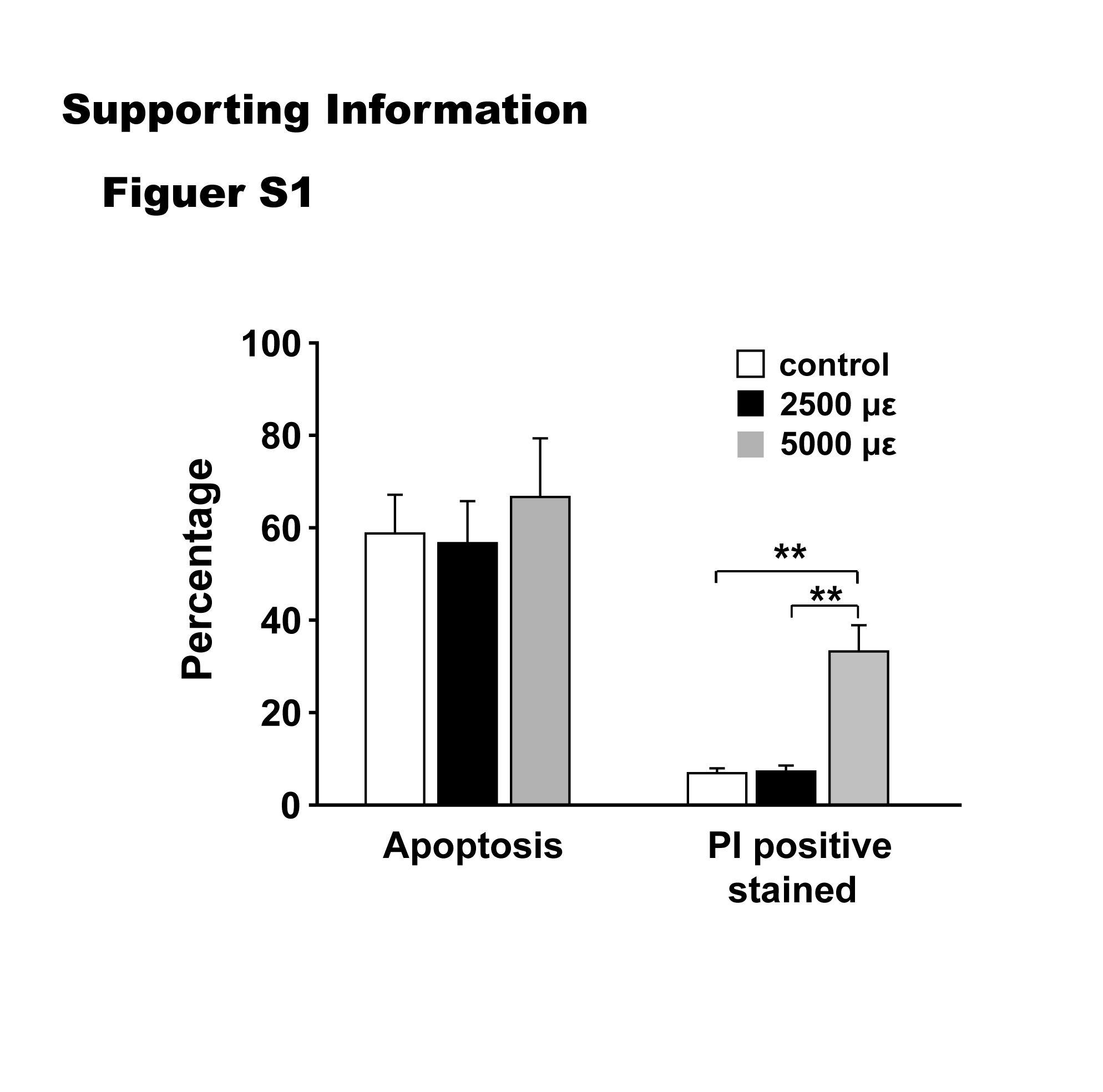

Supplement: Figure S1 — Apoptosis percent and PI positive stained percent of MC3T3-E1 cells. With Apoptosis Assay Kit containing Annexin V labeled with fluorescein isothiocyanate (FITC) and propidium iodide (PI), using fluorescence microscope and flow cytometry, apoptosis percent and PI positive stained percent of MC3T3-E1 cells subjected to different mechanical strain for 3 days were assayed. The apoptosis percent and PI positive stained percent of the cells exposed to 5000 µε were both higher than other groups (0 µε and 2500 µε). The elevation of apoptosis percent was not evident. All data represent the mean ± SD of at least three biological replicates; * P<0.05, ** P<0.01, between the indicated groups. (TIF) [file pone.0035709.s001.tif]

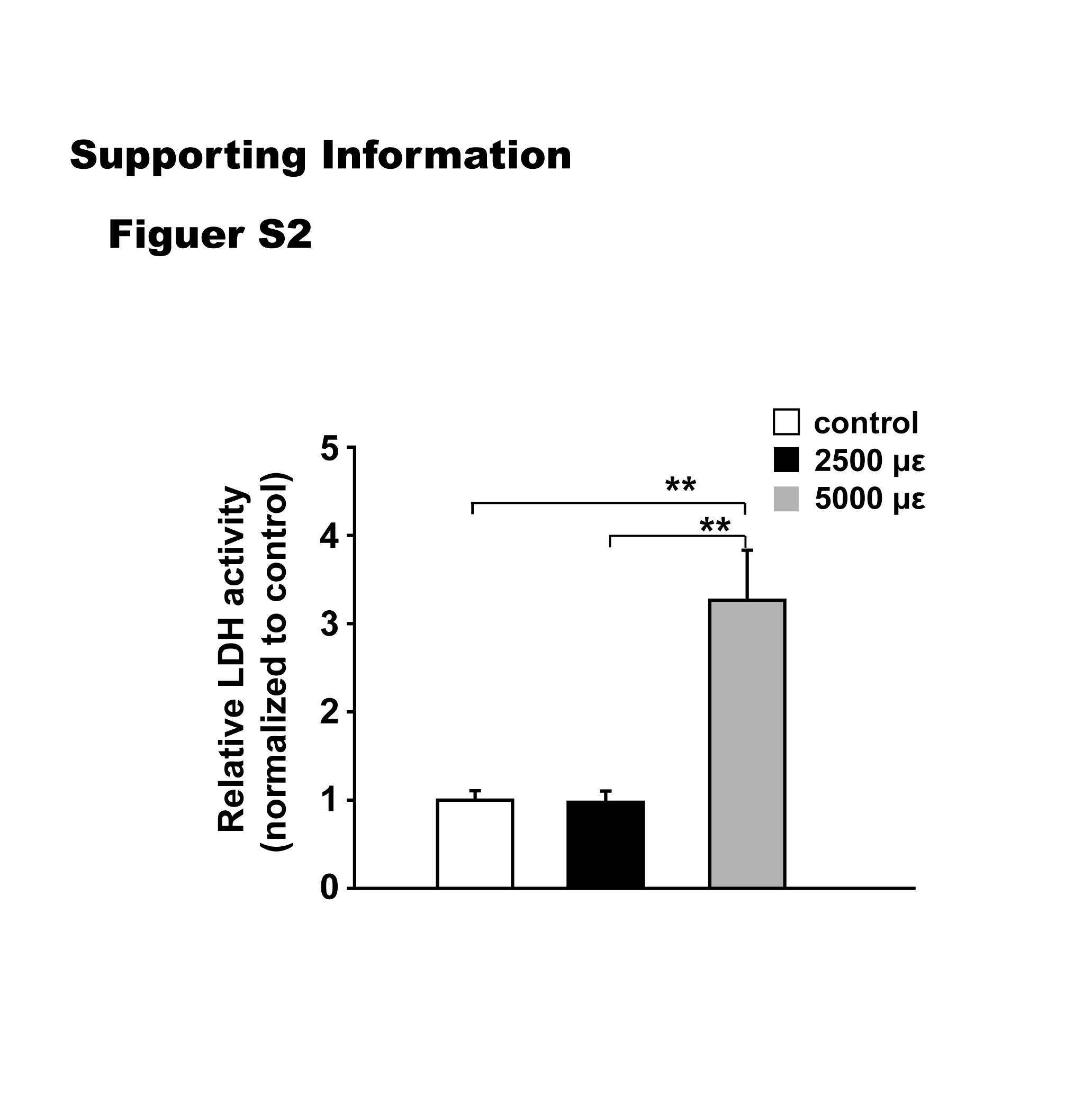

Supplement: Figure S2 — Lactate dehydrogenase (LDH) activity in MC3T3-E1 cells, culture media. With LDH activity assay Kit, using spectrophotometer, the LDH activity in the cells, culture media was assayed. when MC3T3-E1 cells subjected to different mechanical strain for 3 days, the relative LDH activity in the culture media of the cells exposed to 5000 µε were both higher than other groups (0 µε and 2500 µε). All data represent the mean ± SD of at least three biological replicates, * P<0.05, ** P<0.01, between the indicated groups. (TIF) [file pone.0035709.s002.tif]

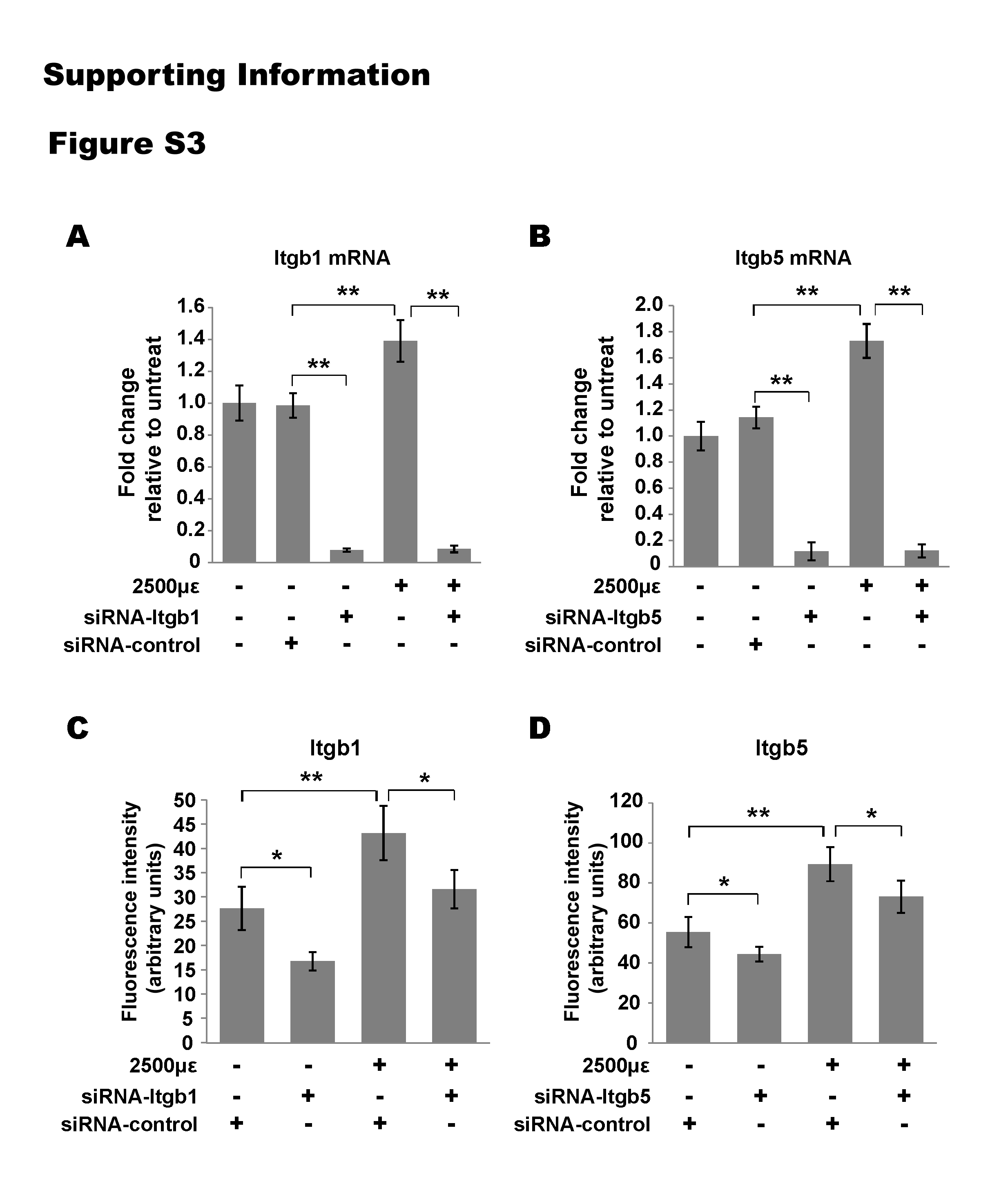

Supplement: Figure S3 — Silencing of Integrin β1 or Integrin β5 by specific siRNA respectively. (A and B) The knockdown efficiency of Integrin β1 (A) or Integrin β5 (B) mRNA was verified by qPCR. (C and D) The knockdown efficency of Integrin β1 (C) or Integrin β5 (D) protein was verified by immunofluorescence. All data represent the mean ± SD of at least three biological replicates; * P<0.05, ** P<0.01, between the indicated groups. (TIF) [file pone.0035709.s003.tif]

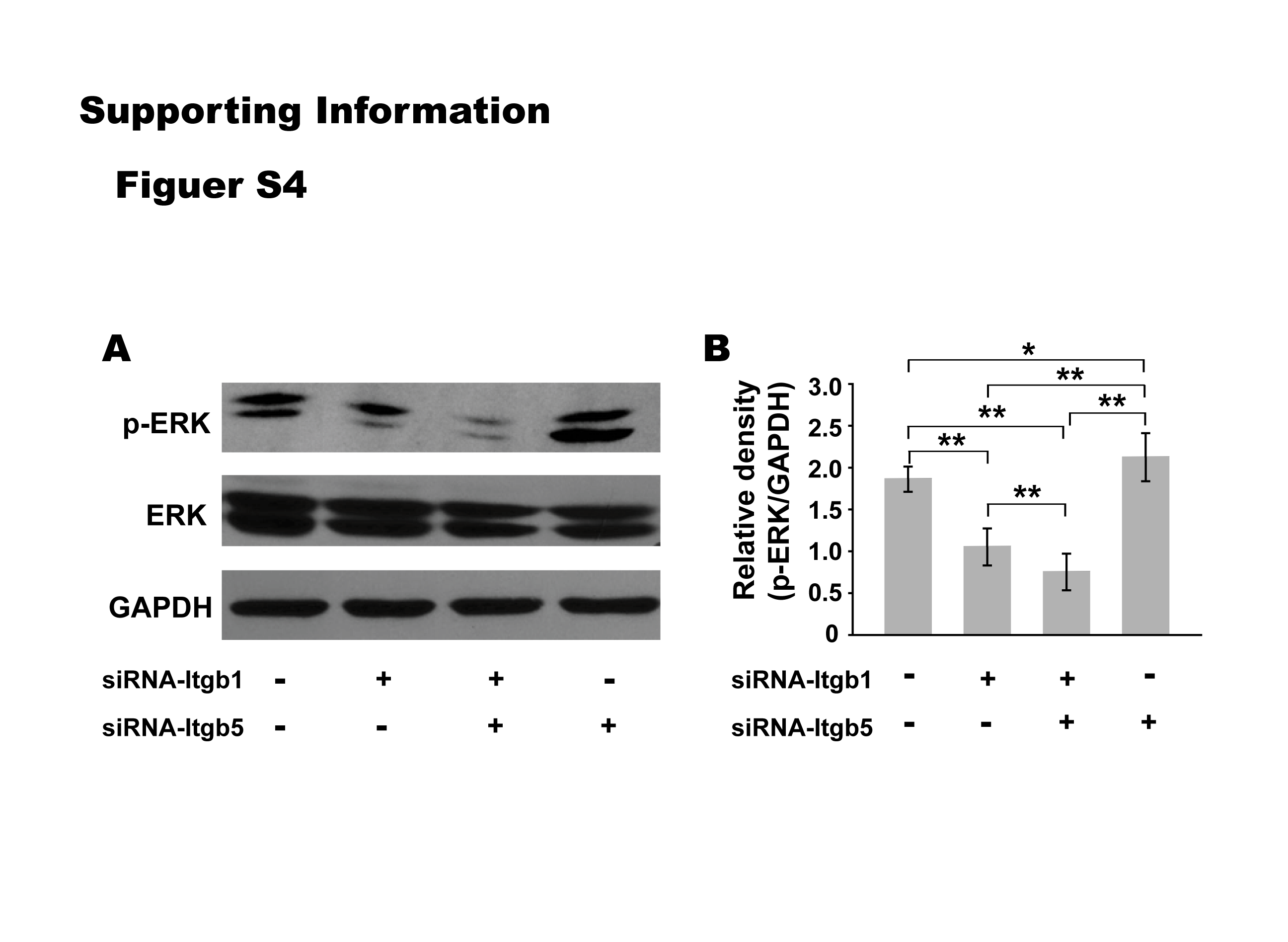

Supplement: Figure S4 — The protein expression of ERK and ERK-phosphorylations in unstrained cells. The MC3T3-E1 cells, protein levels of ERK and ERK-phosphorylations in different groups {Knockdown of Integrinβ1 (Itgb1), Integrinβ5 (Itgb5) or both simultaneously with siRNA transfection} were detected by Western blotting with anti-ERK1/2 and anti-p-ERK1/2. GAPDH was used as an internal control. All data represent the mean ± SD of at least three biological replicates; * P<0.05, ** P<0.01, between the indicated groups. (TIF) [file pone.0035709.s004.tif]

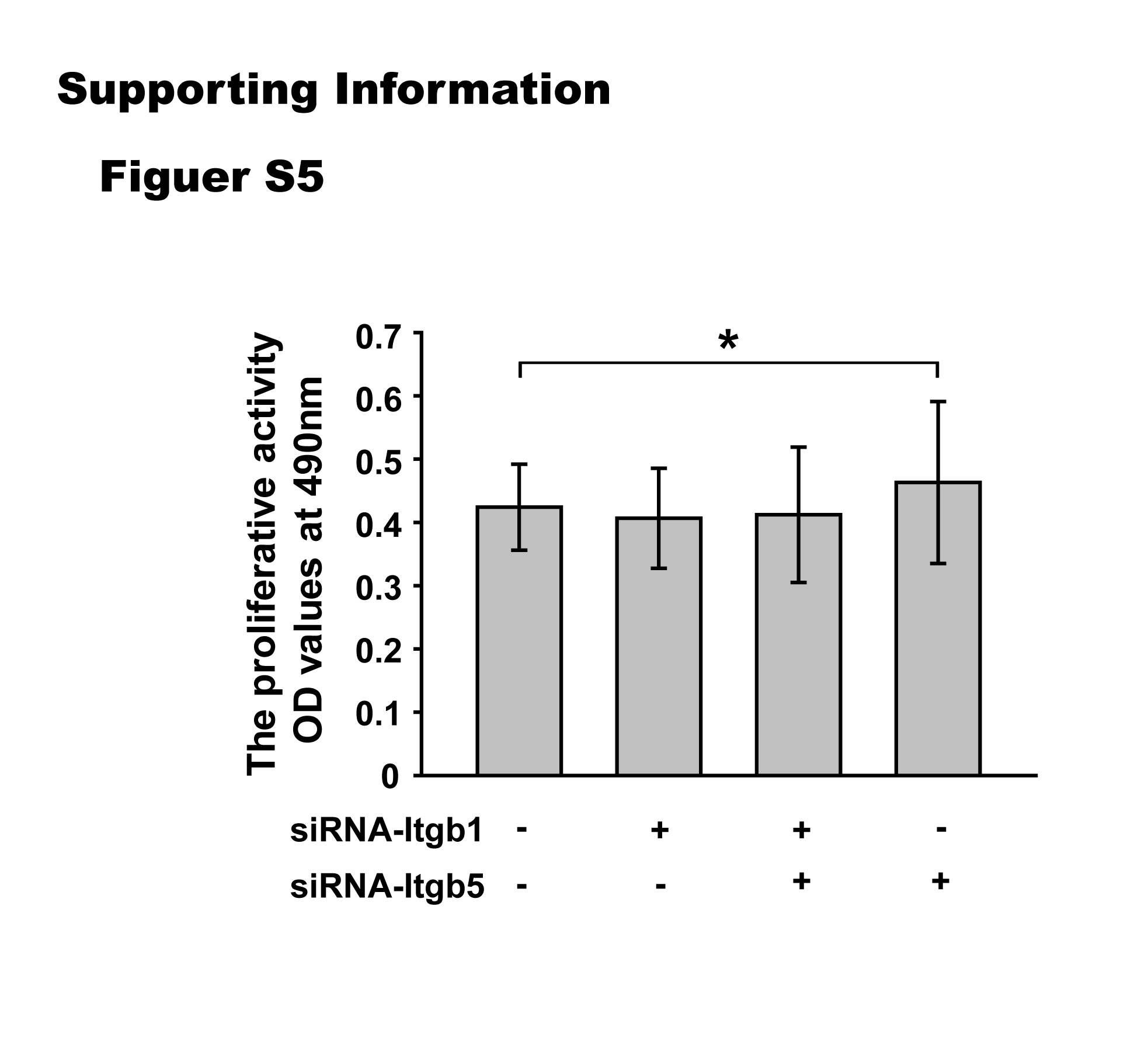

Supplement: Figure S5 — The proliferative activity of unstrained MC3T3-E1 cells. The proliferative activity of the cells in different groups {Knockdown of Integrinβ1 (Itgb1), Integrinβ5 (Itgb5) or both simultaneously with siRNA transfection} were detected by MTT assay. All data represent the mean ± SD of at least three biological replicates; * P<0.05, ** P<0.01, between the indicated groups. (TIF) [file pone.0035709.s005.tif]
