# Supplementary material for: Mechanical Strain Regulates Osteoblast Proliferation through Integrin-Mediated ERK Activation
Source: PLoS One. 2012 Apr 23;7(4):e35709. doi: 10.1371/journal.pone.0035709 (PMC3335094; doi:10.1371/journal.pone.0035709)
Supplement: Table S1 — Sequences of primers used for qRT–PCR. (DOC) [file pone.0035709.s006.doc]

**Table S1. Sequences of primers used for qRT–PCR**

| GB accession no | Gene symbol |  | Sequence (5´- to -3’) | Amplicon size |
| --- | --- | --- | --- | --- |
| X02454 | KrasF | F | AGATGTGCCTATGGTCCTGGT | 106bp |
|  |  | R | AATGAACGGAATCCCGTAACT |  |
| X52685 | Prkca | F | ACCAAGAAGAGGGCGAATACT | 120bp |
|  |  | R | TGATGACTTTGTTACCAGCAG |  |
| BC028260 | Map2k5 | F | GGAGAGTTCTCGGAGCCGTT | 150bp |
|  |  | R | GCACACCCACATGGACACCAC |  |
| BC066000 | Ppp3cb | F | CAGGGAGGAGAGTGAAAGCGT | 289bp |
|  |  | R | AGTCCCGTGGTTCTCAGTGGT |  |
| AC124505 | MAPK3 | F | CCTGGAAGCCATGAGAGATGT | 104bp |
|  |  | R | AGTAGCAGATGTGGTCATTGC |  |
| Y00769 | ITGB1 | F | GCAACGCATATCTGGAAACA | 140bp |
|  |  | R | CAAAGTGAAACCCAGCTACC |  |
| BC058246 | ITGB5 | F | TCCTGCTTCGAGAGTGAGT | 137bp |
|  |  | R | CCTGCGTGGCATTTGCATT |  |
| GU214026 | GAPDH | F | TGCACCACCAACTGCTTAGC | 172bp |
|  |  | R | GGCATGGACTGTGGTCATGAG |  |
